# Exploratory Laparotomy and Abdominal Cancer

**Published:** 1909-07-31

**Authors:** 


### The Hospital

A Journal of

The Medical Sciences and Hospital Administration.

New Series. No. 127, Vol. V. [No. 1199, Vol. XLVI.] SATURDAY, JULY 31, 1909.

### Exploratory Laparotomy and Abdominal Cancer.

A physician has unkindly said that a surgeon's idea of abdominal diagnosis is to "look inside and see." The charter of the Royal College of Physicians of London has a phrase beginning "Whereas medicine doth comprehend surgery," or words to that effect, and there is a sense in which this statement remains as true as when it was indicted. For the surgeon of to-day is a good physician who has educated his fingers and established an imperturbable control over what is vulgarly—and not altogether inaccurately—known as his "nerve." It must be borne in mind, then, that an exploratory or diagnostic laparotomy, the "test-laparotomy" of the Germans, is not undertaken to find out what is the matter with the patient. It is employed as a means of supplying certain additional data without which the internist finds himself unable to proceed to a rational diagnosis; it is the last step, not the first, in demonstration of the lesion, and, moreover, it is the open road to treatment. However trite all this may sound, it would seem that unless kept constantly before us there is danger of too ready and too careless resort to opening the abdomen. Anæsthetics and antiseptics have removed the wholesome restraint of serious risk: but it would be a thousand pities if too light-hearted employment of an invaluable method should imperil the chances of teaching the public a proper appreciation of its importance and necessity.

It is, however, for a more extended use of exploratory laparotomy that we wish to enter a plea. It is only too well known that the early stages of cancerous processes in the gastro-intestinal tract do not manifest themselves by demonstrable or even recognisable symptoms. On the other hand, it is equally well known that only in the early stages can effective measures be taken. Nearly a hundred successful partial gastrectomies are now on record, and it has long been common knowledge that enterectomies may effect cures in cases of intestinal new growth; but it has also been proved conclusively that the fancied immunity of these neoplasms from glandular metastasis—at any rate until a late stage—has been overstated; and there is all the more urgency for early surgical interference. The great majority of cases of intestinal new growth eventually reach the stage of intestinal obstruction, and every hospital surgeon has time after time to perform urgency operations in cases that might have been "cured" by operations of expediency at the proper time.

Unfortunately "the proper time" is generally a time when the patient does not realise he is gravely ill; at a time, in fact, when he is not gravely ill, so far as symptoms go; when perhaps it is nothing more than the development of progressively severe constipation, an occasional attack of looseness of the bowels, some abdominal pain of colicky character, slight distension, borborygmi, and perhaps an attack of vomiting or nausea, or some such symptom-complex, which suggests to the medical adviser the possibility of actual narrowing of the bowel. Or, again, there may be a train of slight symptoms suggestive of gastric disorder; but whilst here something may often be felt, it is seldom so with intestinal new growth.

Still, the surgeon is not to be called in on mere suspicion or surmise. But when the reports of chemist, clinical pathologist, and radiographer have aroused suspicion, his early assistance is imperative. It is of the utmost importance that the surgeon setting out upon an exploratory laparotomy should have every detail of the existing evidence at his finger ends. It is by no means easy to explore the whole length of the intestinal canal through a median laparotomy wound, and small tumours may be missed unless the most systematic and careful examination be made. If, for example, there has been suspicion of obstruction in the upper part of the small intestine, and upon opening the abdomen the colon and duodenum are found closely bound to the gall-bladder by tight adhesions, it becomes of the first importance for the operator to know whether the vomit has contained bile. If bile has never been present, it is improbable that the stricture is above the papilla, and the surgeon must look elsewhere for the actual cause; or at least he should satisfy himself that there is no other obstruction lower down before he closes the abdomen. If he has been wise, and circumstances have permitted, he will have observed previously the passage of a bismuthised meal with the aid of the $x$-rays and screen.

But such examples might be multiplied indefinitely. Moreover, when direct inspection has done its part in settling the diagnosis, it does not mean merely that curiosity is satisfied. Where certainty is reached, it very often means prompt cure by the only available means, removal. Even where doubt is not dispelled the circumvention of some mechanical disability or the avoidance of the "detestable colostomy" may still be feasible. The point is that many patients have far more to gain than to lose by submitting to operation, and it becomes the duty of medical men to put this before them at the right time. When obstruction has set in the patient does not need to be told that an operation is necessary: he knows it himself. And if he should refuse an exploratory operation at a time when cure is possible, it is not the doctor's fault if time should at last convince the unhappy patient that he has thrown away his only chance of recovery against the advice of his medical attendant. If, on the other hand, no dire results follow exclusion of the surgeon, the doctor's reputation will not suffer, if he has explained the matter from the first in lucid terms and the right spirit.

